# Decreasing blood wastage during ex vivo lung perfusion recovery through utilization of thermal control technology

**DOI:** 10.1111/jocs.17147

**Published:** 2022-11-09

**Authors:** Blaine Johnson, Jamie Bucio, Christopher Salerno, Valluvan Jeevanandam, Tae Song, Geoffrey Wool

**Affiliations:** ^1^ Perfusion Services UChicago Medicine Chicago Illinois USA; ^2^ Department of Surgery, Section of Cardiac Surgery University of Chicago Chicago Illinois USA; ^3^ Department of Pathology University of Chicago Chicago Illinois USA

**Keywords:** blood usage, blood wastage, ex vivo lung perfusion, lung transplantation, organ care system, quality improvement

## Abstract

**Background:**

The Organ Care System (OCS) is a revolutionary ex vivo organ perfusion technology that can potentially expand the organ retrieval range. The OCS Lung device uses packed red blood cells (pRBC) with a proprietary solution. We report the ability to reduce blood waste during this procedure by using a thermal packaging solution in conjunction with the OCS platform.

**Methods:**

We retrospectively reviewed all OCS Lung recoveries performed by our recovery team, using pRBCfrom May 2019 to January 2021. Initially, units were stored using passive refrigeration with the Performance cooler at a temperature range of 1–6°C for 4 h. Subsequently, thermal control technology with the ProMed cooler was utilized to maintain the same temperature range for 72 h.

**Results:**

Twenty‐three recoveries were initiated with 63 pRBC. The Performance cooler was used for 8, while the ProMed cooler for 13. 37.5% of pRBC transported with the Performance cooler was used within the validated time range, while 25.0% were used beyond the validated time range based on clinical judgment. In addition, 37.5% of pRBC transported with the Performance cooler were returned to the institution after canceled recoveries with an estimated loss of $1800; the ProMed cooler had no wastage.

**Conclusions:**

This study showed that using an advanced thermal packaging solution facilitates proper storage of pRBC and represents an advancement for extended donor lung preservation. The elimination of blood wastage in this initial study portends ongoing benefits for the limited blood supply and reduced cost.

**Abbreviations**
DBD: donation after brain death
DCD: donation after circulatory death
EVLP: ex vivo lung perfusion
OCS: Organ Care System
OPO: organ procurement organization
pRBC: packed red blood cells
TIC: thermal isolation chamber
VIP: vacuum insulation panel

## 1 INTRODUCTION

Lung transplantation is an effective therapy for patients with end‐stage lung disease.^1^ However, donor availability and donor organ quality continue to be critical challenges to lung transplantation. More than 80% of donor lungs are potentially injured or infected and are considered unsuitable for transplantation.^2^ Significant improvements in patient outcomes have been made related to changes in donor selection, organ preservation, perioperative management, and better treatment of postoperative complications.^3^ Ex vivo lung perfusion (EVLP) is a significant advancement in donor lung preservation that can expand the number of lungs available for transplantation and decrease the waiting time for recipients.^4^, ^5^ The Organ Care System (OCS) (TransMedics) is one type of revolutionary EVLP technology that can expand the organ retrieval range. The OCS Lung is currently the only United States Food and Drug Administration‐approved, portable, normothermic lung perfusion system used to recover standard and expanded criteria donor lungs.^6^, ^7^, ^8^

Current EVLP systems use a variety of perfusates.^9^, ^10^ OCS Lung uses packed red blood cells (pRBC) diluted in OCS Lung solution. OCS Lung solution is a high oncotic, colloid‐based extracellular low potassium solution. The use of pRBC is a logical choice compared to whole blood as it limits viscosity, maximizes availability, and reduces interference with other transplant teams.^9^ When selecting a cooler to transport pRBC units for OCS Lung recoveries, several factors were important. The ideal transport cooler would be durable, and lightweight, offer easy and quick conditioning, and maintain the 1–6°C storage temperature required by the Association for the Advancement of Blood & Biotherapies Standards for a long duration. Prehospital programs have widely utilized thermal control technology for helicopter emergency medical services.^11^, ^12^, ^13^ Compared to passive refrigeration, thermal control technology has also been used to transport temperature‐sensitive medications.^14^, ^15^ However, thermal control technology in conjunction with EVLP has not been researched. With an increase in the utilization of EVLP (over greater distances), viable blood storage and transportation solutions will become an essential component of EVLP lung recovery.

We describe the processes and standard work governing safe storage, transportation, and stewardship of blood products within an OCS Lung program and review quality improvement data from all recoveries examining usage, demographics, and outcomes.

## 2 MATERIAL AND METHODS

### 2.1 Study design

In this single‐center retrospective review, we evaluated all OCS Lung recoveries performed by our recovery team between May 2019 and January 2021. Recovery data, including transportation and logistical details, was obtained from institutional OCS Lung run sheets. Blood traceability and usage data were reviewed using the Sunquest Blood Bank database (Sunquest Information Systems), with specific consideration given to pRBC issuance, transfusion, and waste data for all recoveries. Temperature monitoring data was collected using an ESCORT iLog temperature logger (Cryopak Verification Technologies). According to Chicago Medicine institutional policy, this project received a formal Determination of Quality Improvement status. This initiative was deemed not to be human subject research and was therefore not reviewed by the Institutional Review Board.

### 2.2 Perfusate requirements for OCS Lung

**Figure 1:**
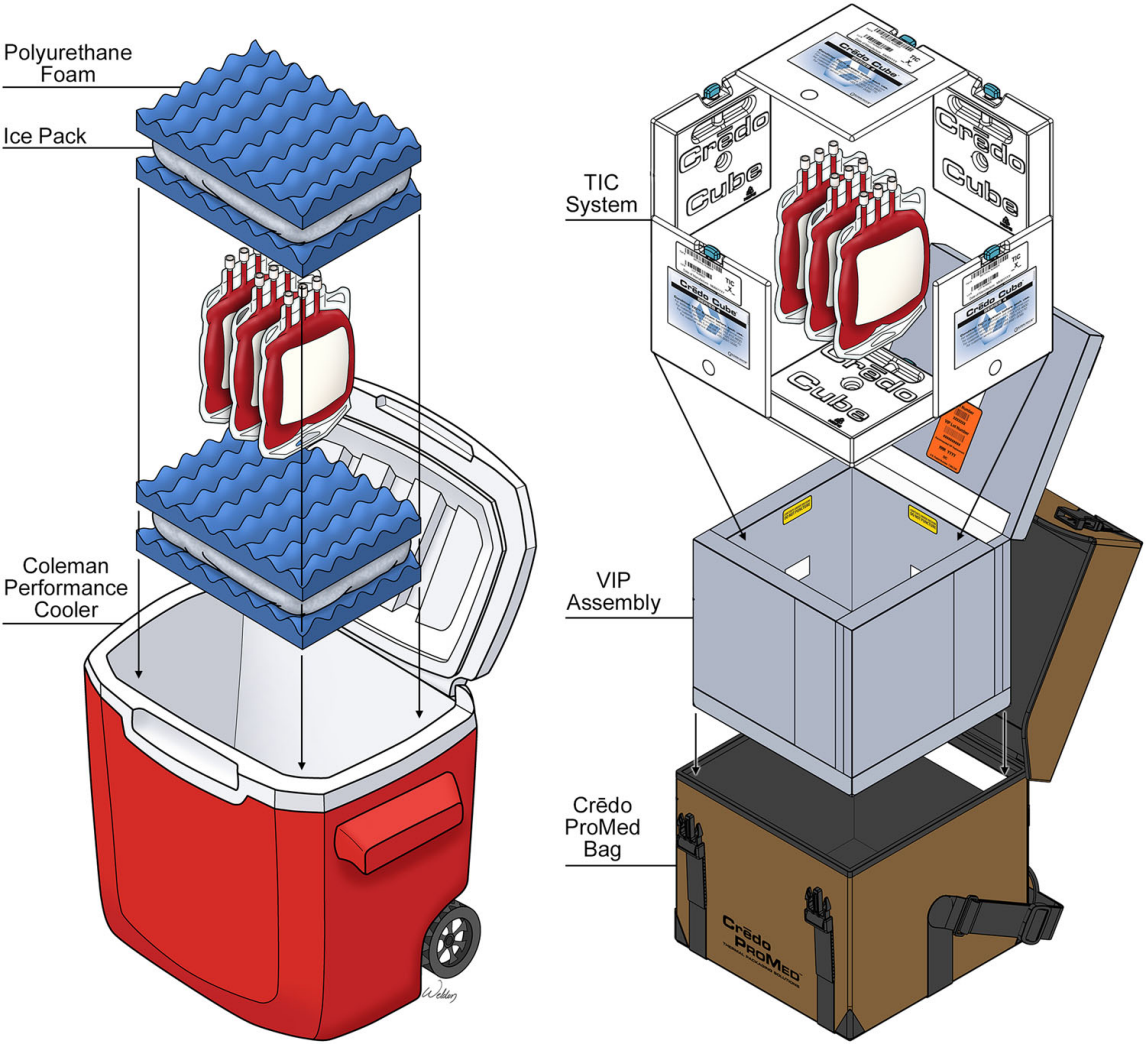
Comparison of the performance and ProMed cooler. Blood bank personnel prepare and seal each blood cooler with three packed red blood cells units and the temperature logger inside. Figure adapted with permission from Peli BioThermal. Printed with permission from UChicago Medicine.

Three units of leukocyte‐reduced pRBC are transported and used as EVLP perfusate (Figure 1). These pRBC units are Cytomegalovirus (CMV) seronegative when indicated (both recipient and donor CMV seronegative). With adequate time for recipient type and screen and ABO verification, the primary preference is to utilize pRBC units released by the blood bank at our institution. These units are ABO compatible with the donor and cross‐match compatible with the recipient. If cross‐matched blood is unavailable before the departure of the recovery team, our secondary preference is to ask the donor site to provide pRBC units. In that case, the organ procurement organization coordinator can provide leukoreduced, pRBC units typed and screened against the donor in the donor operating room. If this second option is unavailable, our tertiary preference is to utilize three of our institution's pRBC units: Emergency‐release leukoreduced group O+.

### 2.3 Storage system

**Figure 2:**
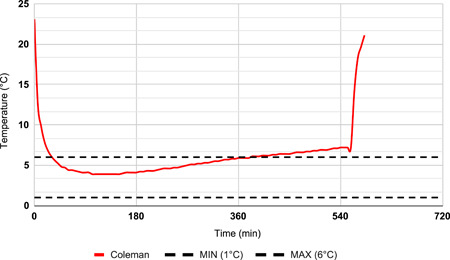
Representative temperature log for performance validation. Initial temperatures were above 6°C as the cooler and data logger were not conditioned at refrigerator temperatures. End of storage excursion at 378 min.

**Figure 3:**
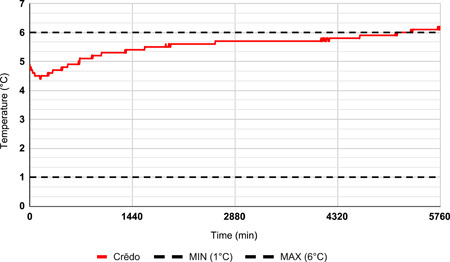
Representative temperature log for the ProMed validation. Temperature excursion at 5340 min.

During the first 6 months, a Coleman Performance 28‐Quart Wheeled Cooler (Coleman Company) was packed with corrugated foam and gel packs stored at −30°C. This cooler was validated to maintain three pRBC units at a temperature range of 1–6°C for 4 h (Figure 2). During the following 15 months, three pRBC units were stored using a Crēdo ProMed 4‐Liter Series 4 Cooler (CR04A4472; Pelican BioThermal) packed with thermal isolation chamber (TIC) panels with phase change material and vacuum insulation panel (VIP) components, which is validated to maintain a temperature range of 1–6°C for 72 h (Figure 3).

### 2.4 Administration process

All Performance coolers were prepared solely according to institutional validation procedures as the manufacturer does not specify a validation procedure. Insulated ice packs are conditioned in a −30°C freezer. Two ice packs are placed between two blue corrugated foam panels to pack the cooler. The foam‐ice pack‐foam assembly is then placed at the bottom of the cooler. The bagged units are then placed in the payload area, and another foam‐ice pack‐foam assembly is placed on top. The container lid is then closed and issued for a validated 4 h storage time frame.

All ProMed coolers are prepared according to institutional validation of the manufacturer's procedure. The conditioning begins with six TIC gel panels set in a −30°C freezer for a minimum of 24 h. Before loading the cooler, the TIC panels are placed in a walk‐in refrigerator maintained at 1–6°C for a minimum of 1 h for proper conditioning. Next, five TIC panels form the base and sides that line all the cooler's interior surfaces. Once the TIC panels are staged and adequately placed inside the VIP assembly, the temperature data logger and bagged units are placed in the payload area, and the sixth and final TIC panel is placed on top. Finally, the cooler's outer insulator lid is closed, secured with two adjustable latches, and issued for a validated 72 h storage time frame.

### 2.5 Quality improvement process

**Figure 4:**
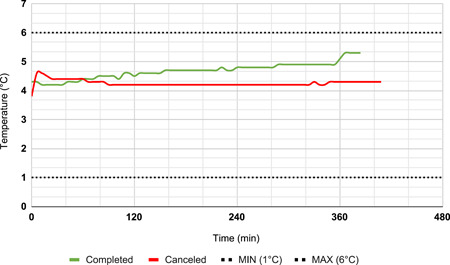
Example temperature logs during ProMed lung recoveries. Temperature measurement of the ProMed cooler during a completed and canceled organ recovery.

To compare the effect that the new ProMed cooler and the original Performance cooler had on blood product wastage, data on total pRBC units issued and transfused and total pRBC wasted were compared: (1) from May 1, 2019, to November 15, 2019 (before the implementation of the revised cooler) and (2) November 16, 2019, to January 31, 2021 (after implementation of the revised cooler). Outcome and logistical metrics for each recovery were also recorded. While no temperature log data was available for the Performance cooler, detailed temperature log data were collected every 6 min for the ProMed coolers due to the long duration of validation (Figure 4).

The primary outcomes were total units of pRBCs issued or wasted and cost analysis of blood product waste. Estimation of total institutional acquisition costs for a single unit of pRBCs ($200) was used for cost analysis.

## 3 RESULTS

### 3.1 Recovery demographics

Baseline recovery demographics are detailed in Table 1. Seventeen recoveries (73.9%) were completed successfully, while six recoveries (26.1%) were canceled. Donations after brain death accounted for 21 recoveries (91.3%). A total of 18 recoveries (78.3%) were transported by air to the donor site, while four recoveries (17.4%) traveled using ground transportation. One recovery (4.3%) occurred within our institution and did not require specialized blood transportation. The mean distance traveled by air transportation was 364.89 ± 297.06 miles compared to 34.13 ± 36.98 miles by ground. The mean recovery time for completed recoveries was 523 ± 120 min.

**Table 1:** Baseline recovery characteristics

Variables	All recoveries	Performance^a^	ProMed^a^
Outcome			
Completed	17 (73.9)	5 (62.5)	11 (84.6)
Canceled	6 (26.1)	3 (37.5)	2 (15.4)
Donor type			
DBD	21 (91.3)	7 (87.5)	12 (92.3)
DCD	2 (8.7)	1 (12.5)	1 (7.7)
Transportation^b^			
Air	18 (78.3)	4 (50.0)	12 (92.3)
Ground	4 (17.4)	3 (37.5)	1 (7.7)
Distance,^b^ mile			
Air	364.89 ± 297.06	265.25 ± 144.86	361.85 ± 316.95
Ground	34.13 ± 36.98	43.17 ± 39.5	7 ± 0
Duration, min			
Completed	523 ± 120	491 ± 179	540 ± 78

*Note*: Values are expressed as *n* (%); mean ± standard deviation.

Abbreviations: DBD, donation after brain death; DCD, donation after circulatory death.

^a^ Excludes recovery using organ procurement organization blood.

^b^ Excludes recovery at institution.

### 3.2 Cooler system utilization

A total of 63 pRBC units (91.3%) were issued from the recipient site with 100% traceability. The Performance cooler was used for eight recoveries (34.8%) that required 24 pRBC units during the first 6 months. Following this time, 13 recoveries (56.5%) were completed with the ProMed cooler which required 39 pRBC units. Overall, two recoveries (8.7%) required pRBC units from the donor site's organ procurement organization (six pRBC units total).

### 3.3 Disposition of blood units

The recovery outcomes and duration of use were not clinically significant between groups (Tables 2 and 3). A total of nine pRBC units (37.5%) transported during completed recoveries with the Performance cooler were used at the donor site within the 4 h validation time with a mean duration of 152.50 ± 13.44 min. In comparison, six pRBC units (25.0%) transported with the Performance cooler were used based on clinical judgment beyond the 4 h validation with a mean duration of 306.67 ± 15.28 min. All 33 pRBC units (84.6%) transported during completed recoveries with the ProMed cooler were used within the 72 h validation time with a mean duration of 271.27 ± 60.03 min. A total of nine pRBC units (37.5%) transported with the Performance cooler were returned to the institution after canceled recoveries and wasted due to time deviation. A total of six pRBC units (15.4%) transported with the ProMed cooler were returned to the institution after canceled recoveries and all were returned to stock for reallocation.

**Table 2:** Blood unit data

Variables	All units	Performance^a^	ProMed^a^
Blood source site			
Recipient	63 (91.3)	24 (100)	39 (100)
Donor	6 (8.7)	‐	‐
Used at donor site^a^			
Within validation	42 (66.7)	9 (37.5)	33 (84.6)
Beyond validation	6 (9.5)	6 (25.0)	0
Returned to institution^a^			
Wasted for time deviation	9 (14.3)	9 (37.5)	0
Returned to stock	6 (9.5)	0	6 (15.4)

*Note*: Values are expressed as *n* (%).

^a^ Excludes recovery using organ procurement organization blood.

**Table 3:** Cooler performance data

Variables	All units	Performance^a^	ProMed^a^
Duration, min	263.06 ± 67.12	245.00 ± 85.39	271.27 ± 60.03
Within validation	253.00 ± 70.77	152.50 ± 13.44	271.27 ± 60.03
Beyond validation	306.67 ± 15.28	306.67 ± 15.28	N/A

*Note*: Values are expressed as mean ± standard deviation.

a Excludes recovery using organ procurement organization blood.

### 3.4 Cost analysis

Nine pRBC units (37.5%) were transported with the Performance cooler and wasted due to time deviation (Table 4). When extrapolated, that wasted blood product represents an annual replacement cost of approximately $3600 for 18 units to be used over nine recoveries. In comparison, the ProMed cooler allowed longer maintenance of proper pRBC storage temperature and eliminated blood wastage costs.

**Table 4:** Blood unit cost analysis

Cooler Type	Units issued	Units used in prime	Returned units wasted	Wastage percentage	Estimated wastage cost
Performance	24	15	9	37.5%	$1800
ProMed	39	33	0	0%	$0

At least two of each type of cooler were purchased to allow continued OCS Lung recovery operation. For example, if one of the coolers were to be damaged, lost, or in use. Additionally, a set of data loggers was purchased to accompany each ProMed cooler. It is noted that the acquisition cost for ProMed coolers was significantly higher than the Performance coolers (Table 5). However, when the acquisition costs ($1295) are compared to the ability to eliminate blood wastage ($3600), the ProMed coolers were found to far surpass their breakeven cost at 1 year, with a savings of $2305. During the same 1‐year period, the Performance cooler led to a loss of $3690.

**Table 5:** Cooler acquisition cost analysis

Cooler type	Cost of cooler set	Cost of data loggers	Total cost of acquisition
Performance	$90	$0	$90
ProMed	$790	$505	$1295

### 3.5 Temperature monitoring

A total of five ProMed recoveries (38.5%) were retrospectively identified to have temperature excursions outside the allowable 1–6°C range. The median duration of temperature deviation was 60 min with a range of 6–198 min. 80% of the temperature excursions were noted immediately after cooler preparation and represent inadequate temperature conditioning of cooler components. In such cases, the temperature trend subsequently entered and remained in the allowable 1–6°C range. Therefore, these deviations are favored not to represent a temperature change of the pRBC product. The median high‐temperature excursion of all samples was 6.3°C, while the median low‐temperature deviation was 0.1°C.

## 4 DISCUSSION

Our results are the first reported use of thermal control technology to reduce blood wastage in conjunction with EVLP utilizing OCS Lung. Compared to our previous passive refrigeration standard using the Performance cooler, the thermal control technology with the ProMed cooler offered superior conditioning and transportation capabilities. In addition, the ProMed cooler offers an 18‐fold longer validated time to maintain the required 1–6°C blood storage temperature than our previous cooler. In keeping with this extended period of stable storage temperature, wastage dropped from 37.5% of pRBC units to 0% when the ProMed cooler was implemented for OCS Lung. While limited, these findings suggest significant early improvement in blood wastage for lung recovery.

Previous publications have demonstrated the superiority of thermal control technology over passive refrigeration for medication and emergency blood transportation. Clancy et al. found that the industry‐accepted passive refrigeration was ineffective for temperature‐sensitive medication transport and resulted in significant temperature excursions for trip durations lasting greater than 3 h.^15^ Utilizing thermal control technology, Krook et al.^11^ reported only 1.2% of unit wastage during a 4‐year review of prehospital blood transfusion within a Canadian helicopter emergency medical service. Blood is a valuable resource that is often in short supply.^16^, ^17^ The coronavirus disease 2019 (COVID‐19) pandemic has adversely affected blood donation due to social distancing and resulted in the cancellation of blood drives, creating an increasing strain on the national blood supply.^18^, ^19^, ^20^ Hospitals should routinely monitor blood product wastage as a quality indicator, investigate factors contributing to the wastage, and establish systems to reduce wastage.^21^

Blood wastage accounted for 14.2% of the total units issued for lung recovery by our institution's blood bank. All these wasted units were transported with the Performance cooler. Based on organ transplantation's essential and emergent nature, these pRBC units were stored longer than their cooler's validated storage duration, resulting in temperature excursions. Our multidisciplinary team of perfusionists and blood bank personnel have become more familiar over time with the storage and stewardship processes, demonstrating the success of quality improvement practices. To date, all unused pRBC units from canceled recoveries using the ProMed coolers have been successfully returned to our blood bank within the validated storage timeframe and reallocated for future use. In addition to avoiding the wastage of a valuable resource, the ability of thermal control technology to maintain a stable temperature long‐term resulted in a 5‐year cost savings of approximately $18,000.

Ultimately, thermal control technology with continuous temperature monitoring improves quality measurement and patient safety. Using the iLog temperature logger during the recovery phase allows for real‐time, data‐driven clinical decision support. Additionally, every data set is retrospectively reviewed for quality improvement and assurance. Returned units are quarantined until the temperature data logger can be reviewed to ensure proper temperature compliance throughout transportation. At the same time, process improvements for administered units, such as inadequate conditioning time, can be identified and addressed.

The implementation of thermal control technology had no impact on operational efficiency. There is no additional storage or freezer equipment requirement as the TIC gel panels are stored in existing refrigerators or freezers at our institution's blood bank. However, the conditioning process required for TIC gel panels before issuance required a minimal workflow change for the blood bank technologists.

The ProMed cooler is constructed to protect medical materials such as blood and commercial bio‐pharma products, with relatively easy conditioning of the modular TIC system panels. However, periodic inspection of the VIP lid and base surfaces is imperative as this innovative technology is highly effective only if they hold an internal vacuum. Therefore, the manufacturer recommends the replacement of the VIP every 5 years.

These key features, along with other findings of Martin et al., were the basis of our institution's selection of the ProMed cooler for use with both OCS Lung recovery and the Aeromedical Network.^22^

In the future, a multicenter approach to thermal control technology should be further explored and investigated in conjunction with other ex vivo organ perfusion platforms, such as the OCS Heart and Liver, which recently received approval from the United States Food and Drug Administration.

## 5 LIMITATIONS

There are some limitations to this study. The study sample size is small, but it should be noted that at the time of this study, only 16 institutions in the United States were utilizing OCS Lung for EVLP, with Loor et al.reporting 87% of eligible lungs transplanted with OCS Lung.^7^ pRBC units transported with the Performance cooler did not collect temperature log data. Therefore, actual temperature deviations could not be accurately assessed. Further studies are needed to investigate the clinical benefit of utilizing this technology concerning patient outcomes.

## 6 CONCLUSION

This observational study presents an approach to utilizing thermal control technology in conjunction with EVLP to decrease blood wastage during lung recovery. Ongoing quality improvement initiatives and interdepartmental collaboration help strengthen the process. Our results support the use of thermal control technology to transport blood for use in EVLP. These findings can also apply to other ex vivo organ perfusion platforms. Ex vivo perfusion programs should develop sustainable strategies for blood transportation. Stewardship and standard work processes can be applied to minimize the wastage of precious blood resources.

## AUTHOR CONTRIBUTIONS

Blaine Johnson, Jamie Bucio, Christopher Salerno, Valluvan Jeevanandam, Tae Song, and Geoffrey Wool all collaborated in the writing and editing of this manuscript. All authors contributed to the article and approved the submitted version.

## CONFLICT OF INTEREST

The authors declare no conflict of interest.
